# The CREB and AP-1–Dependent Cell Communication Network Factor 1 Regulates Porcine Epidemic Diarrhea Virus-Induced Cell Apoptosis Inhibiting Virus Replication Through the p53 Pathway

**DOI:** 10.3389/fmicb.2022.831852

**Published:** 2022-03-28

**Authors:** Hongchao Zhou, Yuting Zhang, Jingjing Wang, Yuchao Yan, Yi Liu, Xiaojie Shi, Qi Zhang, Xingang Xu

**Affiliations:** College of Veterinary Medicine, Northwest A&F University, Xianyang, China

**Keywords:** PEDV, CCN1, CREB, AP-1, p53 pathway, apoptosis

## Abstract

Porcine epidemic diarrhea virus (PEDV) infection causes severe diarrhea, dehydration, and high mortality in sick pigs, causing huge economic losses to the pig industry. However, the relationship between cell communication network factor 1 (CCN1) and PEDV infection has not been reported. In this study, we showed that the expression of CCN1 was enhanced by PEDV infection, and we observed that PEDV promotes the CREB and AP-1 activation to promote CCN1 expression. The PKA and p38 inhibitors significantly suppress CCN1 expression, indicating that PEDV-induced CCN1 expression may be through PKA and p38 pathway. Further tests confirmed that CREB and AP-1 are regulated by PKA and p38, respectively. Overexpression of CCN1 decreased the replication of PEDV, whereas knockdown of CCN1 increased the replication of PEDV. We proved that the overexpression of CCN1 increased the phosphorylation level of p53, promoted the expresion of Bax and the cleavage of caspase 9 and caspase 3, and inhibited the production of Bcl-2. CCN1 knockdown decreased the phosphorylation level of p53, inhibited the production of Bax and the cleavage of caspase 9 and caspase 3, and promoted the expression of Bcl-2. The treatment of PFT-α (p53 inhibitor) significantly suppressed the expression of cleaved caspase 9 and caspase 3, leading to the decrease of apoptosis. Together, these studies showed that PEDV promotes the activation of CREB and AP-1 to increase the expression of CCN1. Overexpression of CCN1 promotes apoptosis by elevating p53 protein phosphorylation and inhibits PEDV replication, and knockdown of CCN1 inhibits apoptosis by decreasing p53 protein phosphorylation and promotes PEDV replication. Our study could provide some reference for the molecular mechanisms of PEDV-induced CCN1 induction and supply a new therapeutic target for PEDV.

## Introduction

Porcine epidemic diarrhea (PED) leads to severe diarrhea and dehydration, intestinal villi atrophy, and high mortality of neonatal piglets, causing enormous economic losses for the pig industry. Porcine epidemic diarrhea virus (PEDV), the etiological agent of PED, is an enveloped, single-stranded positive-sense RNA virus with a genome of approximately 28 kb, belonging to the genus *Alphacoronavirus* family Coronaviridae. The PEDV genome has seven open reading frames (ORFs), which encode two non-structural polyproteins (pp1a and pp1b) (Si et al., 2020; Ye et al., 2020), four structural proteins (S, E, M, and N) (Cochrane et al., 2020), and one virulence accessory protein (ORF3) (Lee, 2016). At present, the pathogenesis of PEDV is not clear. Therefore, it is particularly critical to study the interaction between PEDV and cellular proteins to search for the prevention and treatment mechanisms of PEDV.

Apoptosis is a death program performed by cells that can maintain cell homeostasis by clearing damaged and infected cells. It is mainly regulated by the caspase family protein (Ivanisenko et al., 2020). Apoptosis is activated through three different pathways including the extrinsic pathway, the intrinsic pathway, and the endoplasmic reticulum (ER) pathway (Pan et al., 2018). As a cell defense mechanism, apoptosis can be activated through a series of stimuli, including virus infection. For example, PEDV promotes the release of the mitochondrial pro-apoptosis protein AIF to promote cell apoptosis (Kim and Lee, 2014). The p53-PUMA pathway was activated by PEDV, which induced Vero cell apoptosis (Yang et al., 2021). However, the role of the cell proteins in PEDV-induced apoptosis is still unclear.

Cell communication network factor 1 (CCN1) is a cysteine-rich matrix cell protein, which belongs to one of the CCN family members and consists of four conserved domains: insulin-like growth factor–binding protein homologies domain, von Willebrand growth factor C repeat domain, thrombospondin type I repeat domain, and carboxyl-terminal domain–containing cysteine motif (Zhu et al., 2020). As a multifunctional protein, CCN1 regulates diverse cellular processes such as cell adhesion, migration, proliferation, and apoptosis (Chen and Du, 2007; Lau, 2016). CCN1 induces the apoptosis and senescence of cells through the accumulation of reactive oxygen species (ROS) and CCN1 promotes the cleavage of caspase 9 and caspase 3 through p53-dependent Bax activation in fibroblasts (Todorovic et al., 2005; Lau, 2011), but the role between CCN1 and PEDV-induced apoptosis remains unclear. In addition to regulating the function of apoptosis, CCN1 can also be induced by viruses and regulate virus replication. Previous studies have shown that CCN1 induced by oncolytic virus infection can stimulate type I interferon reaction in glioblastoma cells, thus reducing virus replication (Thorne et al., 2014). Instead, the Zika virus by regulating CCN1 promotes ZIKV replication (Sun et al., 2020). However, it is not clear whether the infection of PEDV can regulate the expression of CCN1 and whether CCN1 affects the replication of PEDV.

In this study, we demonstrated that PEDV promotes transcription factors cAMP response element binding (CREB) and Activator protein 1 (AP-1) phosphorylation into the nucleus through protein kinase A (PKA) and p38 pathways. Activated CREB and Activator protein 1 (AP-1) increase the transcriptional expression of CCN1 by combining with the CCN1 promoter. Overexpression of CCN1 can inhibit the replication of PEDV replication and promote cell apoptosis, whereas knockdown of CCN1 promotes the replication of PEDV and inhibits apoptosis. We demonstrated that the levels of phospho-p53(ser15) and phospho-p53(ser20) protein were elevated during CCN1 overexpression, whereas the levels of phospho-p53(ser15) and phospho-p53(ser20) protein were inhibited during CCN1 knockdown. Moreover, the treatment of Pifithrin-α (PFT-α) inhibited CCN1-induced apoptosis, indicating CCN1-induced apoptosis is dependent on the p53 pathway. In conclusion, our study showed that CREB and AP-1 play dominant roles in PEDV-induced CCN1 expression and disclosed the mechanism by which CCN1 regulates cell apoptosis and PEDV replication.

## Materials and Methods

### Cells Cultures, Virus, Virus Titration, and Infection

African green monkey kidney epithelial cells (Marc-145) and Vero cells, PEDV-permissive cell line, were maintained in Dulbecco’s Modified Eagle Medium (Hyclone, Logan, UT, United States) supplemented with 10% fetal bovine serum (PAN-Biotech, Aidenbach, Germany) and culture at 37°C with 5% CO_2_. PEDV variant strain CH/SXYL/2016 was originally isolated from intestinal tract contents suffering from PEDV piglets (Sun et al., 2018). The PEDV strain was amplified and titrated in Marc-145 cells. Briefly, 96-well plates were covered with monolayer Marc-145 and then infected with serially dilute PEDV (10^–1^ to 10^–10^). The virus titer was determined by the Reed-Muench method and transformed into a 50% tissue culture infection dose (TCID_50_). Marc-145 cells were infected with PEDV strain at an multiplicity of infection (MOI) of 1.

### Inhibitors and Antibodies

p38 inhibitor SB203580 (10 μM), c-Jun N-terminal kinase (JNK) inhibitor SP600125 (10 μM), extracellular signal-regulated kinase (ERK) inhibitor PD98059 (5 μM), PKA inhibitor H-89 (5 μM), c-Jun inhibitor SR11302 (5 μM), CREB inhibitor EML-425 (5 μM), and p53 inhibitor PFT-α (10 μM) are all purchased from MedChemExpress (Shanghai, China). All inhibitors were configured with dimethyl sulfoxide (DMSO; Solarbio, Beijing, China). Antibodies against Bax, Bcl-2, caspase 3, caspase 8, caspase 9, FasL, Fas, p53, his, phospho-p53(ser15), phospho-p53(ser46), and phospho-p53(ser20) were purchased from Cell Signaling Technology (Danvers, MA, United States). Monoclonal antibodies against CCN1, c-Jun, phospho-c-Jun, c-Fos, phospho-c-Fos, CREB, and phospho-CREB were obtained from ABclonal (Wuhan, China). Antibodies against PEDV (CH/SXYL/2016) N protein were stored in our laboratory (Xu et al., 2019).

### Construction of the Cell Communication Network Factor 1 Promoter Expression Vectors and Promoter Deletion Mutants

Extraction of genomic DNA from Marc-145 cells using a DNA extraction kit (Tiangen, Beijing, China). The fragment of the CCN1 gene promoter from −1,994 to 203 bp was cloned and inserted into the luciferase reporter vector pGL4.10-basic to generate the CCN1 promoter reporter plasmid (–1994∼203-Luc). A series of CCN1 promoter truncated mutants were constructed and cloned into a pGL4.10-basic vector at the *Kpn*I and *Nhe*I sites. The CCN1 promoters AP-1, SP1, and CREB element deletion mutants were constructed using the primers listed in Table 1 and –985∼203-Luc as a template by overlapping PCR. Then, the constructed mutant sequence was verified and cloned into pGL.4.10-basic vector to generate ΔAP-1-Luc, ΔSP1-Luc, and ΔCREB-Luc. The CCN1 promoter primer and truncated mutants of the CCN1 promoter primer were listed in Table 1.

**TABLE 1:** Primers for truncated sequence of CCN1 promoter^a^.

Primer name^b^	Sequence (5′-3′)^c^
–1994/203-F	CGGGGGTACCTGGATAACAGAGGCAGAA (*Kpn*I)
–1678/203-F	CGGGGTACCCTGTGTACATGTTGGGC (*Kpn*I)
–985/203-F	CGGGGTACCACTGGAATCTGGTTTG (*Kpn*I)
–24/203-F	CGGGGTACCTCCGCCGGCCCATATAA (*Kpn*I)
–1994/203-R	CTAGCTAGCAAGACGCCAACAAGCT (*Nhe*I)
ΔAP-1-F	CGGGGTACCTGGATAACAGAGGCAGAAAAATGTTAA (*Kpn*I)
ΔAP-1-R	CTAGCTAGCAAGACGCCAACAAGCT (*Nhe*I)
ΔAP-1-F_1_^d^	TCCCGGAGAACTCCCCGCGTTCGTTTCCTCTC
ΔAP-1-R_1_^e^	GCGGGGAGTTCTCCGGGATTCCTCACGGATACAGGA
ΔSP1-F	CGGGGTACCTGGATAACAGAGGCAGAAAAATGTTAA (*Kpn*I)
ΔSP1-R	CTAGCTAGCAAGACGCCAACAAGCT (*Nhe*I)
ΔSP1-F_1_	CTCGCGACCCTCCAACCACCATCACCACCATCACA
ΔSP1-R_1_	TGGTTGGAGGGTCGCGAGGTCCAGTTCAGAACTTTG
ΔCREB-F	CGGGGTACCTGGATAACAGAGGCAGAAAAATGTTAA (*Kpn*I)
ΔCREB-R	CTAGCTAGCAAGACGCCAACAAGCT (*Nhe*I)
ΔCREB-F_1_	GCAACACGCGGCGCCTCCGCCGGCCCATATAAAA
ΔCREB-R_1_	GCGGAGGCGCCGCGTGTTGCTCTGTCTGCGCGTT
CCN1-F	TAGAATTCCGCCACCATGAGCTCCCGCATCGC (*Eco*RI)
CCN1-R	CCGCTCGAGGCGTCCCTAAATTTGTGAATGT (*Xho*I)

*^a^Nucleotide 1 represents the site of transcription initiation of the CCN1 promoter.*

*^b^F, forward primer; R, reverse primer.*

*^c^CCN1 promoter gene sequences were downloaded from GenBank.*

*^d,e^Intermediate primer fragment for overlapping PCR overlapping PCR.*

### Overexpression Plasmid Construction and Transfections

Total RNAs were extracted from Marc-145 cells using TRIzol reagent and reverse-transcribed to cDNA using the FastKing RT Kit with gDNase (Tiangen, Beijing, China). The sequence of the CCN1 gene was amplified through using cDNA as a template. The length of the CCN1 gene was confirmed with DNA sequencing and cloned into the pcDNA3.1-his vector to produce pcDNA3.1-CCN1-his. The Marc-145 cells were seeded on a six-well plate. The next day, the pcDNA3.1-CCN1-his were transfected into Marc-145 cells using lipo8000 (Beyotime Biotechnology, Shanghai, China) according to the manufacturer’s instructions. The efficiency of overexpression of CCN1 protein was confirmed by Western blot. The sequences of the CCN1 primer are listed in Table 1.

### Small Interfering RNAs Knockdown and Transfections

Small interfering RNAs (siRNAs) targeting CCN1 protein and a negative control (siNC) were designed and synthesized by biomics (Biomics, Jiangsu, China). Marc-145 cells were grown in a six-well plate, and the siRNA or siNC was transfected using lipo8000 (Beyotime Biotechnology, Shanghai, China) when density was about 70∼80%. At 48 h post-transfection, cells were infected with PEDV and then collected at different time points by Western blot to evaluate the efficiency of knockdown. The sequences of siCCN1 and siNC are listed in Table 2.

**TABLE 2:** Sequences of siRNAs used in this study.

Primer name^a^	Sequence (5′-3′)
siCCN1-1-F	GGCAGACCCUGUGAAUAUADTDT
siCCN1-1-R	UAUAUUCACAGGGUCUGCCDTDT
siCCN1-2-F	GGGAAAGUUUCCAGCCCAADTDT
siCCN1-2-R	UUGGGCUGGAAACUUUCCCDTDT
siCCN1-3-F	CCCGAAUCAGUUAGGUUUADTDT
siCCN1-3-R	UAAACCUAACUGAUUCGGGDTDT
siNC-F	UUCUCCGAACGUGUCACGUTT
siNC-R	ACGUGACACGUUCGGAGAATT

*^a^F, forward primer; R, reverse primer.*

### One-Step Growth Curve Assay

Marc-145 cells were seeded into six-well plates, and, when the cell density reached 90%, PEDV at MOI of 1 was used to infect cells. The cell supernatant was collected, indicating time to detect TCID_50_.

### Quantitative Real-Time PCR

Total mRNA was isolated from Marc-145 cells using TRIzol reagent (Invitrogen, Carlsbad, CA, United States), and 2 μg of total RNA were reverse-transcribed to cDNA using the FastKing RT Kit with gDNase (Tiangen, Beijing, China) according to the manufacturer’s protocol. Quantitative real-time PCR (qRT-PCR) was carried out to detect the expression levels of the specific genes. The relative quantities of mRNA were calculated using the 2^–ΔΔCt^ method. The gene-specific primers for qRT-PCR are listed in Table 3.

**TABLE 3:** List of primers for qRT-PCR.

Primer^a^	Sequence (5′-3′)^b^
PEDV (N)-F	AGATCGCCAGTTTAGCACCA
PEDV (N)-R	GGCAAACCCACATCATCGT
CCN1-F	ACGAGGATAGTGTCAAGGACC
CCN1-R	CAGGGAGCCGCTTCAGT
c-Jun-F	CAGACAGTGCCCGAGATG
c-Jun-R	CCTCATGCGCTTCCTCT
c-Fos-F	TGTGCAACCCACCCTCA
c-Fos-R	CCACTGCTGTAGCCACTCAT
IFN-β-F	ACGGCTCTTTCCATGAGCTAC
IFN-β-R	GTCAATGCAGCGTCCTCCTT
TNF-α-F	TCTGTCTGCTGCACTTTGGAGTGA
TNF-α-R	TTGAGGGTTTGCTACAACATGGGC
IL-6-F	TTACTACAGTGGCAACGAGGATG
IL-6-R	GGAATCAAGGTGCTCAGGTCAT
IL-8-F	GGAACCATCTCGCTCTGTGTAA
IL-8-R	GGTCCACTCTCAATCACTCTCAG
ISG15-F	CACCGTGTTCATGAATCTGC
ISG15-R	CTTTATTTCCGGCCCTTGAT
β-actin-F	CTTAGTTGCGTTACACCCTTTC
β-actin-R	TGTCACCTTCACCGTTCCA

*^a^F, forward primer; R, reverse primer.*

*^b^Monkey gene sequences and PEDV gene sequences were downloaded from GenBank.*

### Western Blotting

The treated Marc-145 cells were washed with PBS twice and then treated with RIPA lysate containing protease inhibitor and phenylmethylsulfonyl fluoride. Protein concentrations were determined using the bicinchoninic acid (BCA) protein quantitative kit (Pierce, Rockford, IL, United States). Equivalent amounts of protein samples were separated by sulfate–polyacrylamide gel electrophoresis and transferred onto polyvinylidene difluoride membranes (Millipore Corp, Atlanta, GA, United States). The membranes were blocked for 1 h in 5% non-fat dry milk or bovine serum albumin at room temperature and incubated overnight at 4°C with the indicated primary antibody. After sufficient washing with PBST, membranes were incubated with appropriate horseradish peroxidase (HRP)-conjugated secondary antibodies at room temperature for 1 h and then subjected to washing. Protein bands were visualized using an ECL reagent (DiNing, Beijing, China).

### Dual-Luciferase Reporter Assays

The Marc-145 cells were seeded in a 12-well dish. The following day, cells were transfected with the constructed plasmids (pRL-TK, pGL-4.10-basic, pGL-4.10-CCN1 promoter, and pGL-4.10-CCN1 mutant promoter) using Lipofectamine 8000 (Beyotime Biotechnology, Shanghai, China) according to the manufacturer’s protocol. At 24 h post-transfection, cells were treated with or without PEDV at MOI of 1 for 48 h. The cells were harvested and lysed and then analyzed by a dual-luciferase reporter assay kit (Promega, Beijing, China) according to the manufacturer’s instructions.

### Flow Cytometry

Marc-145 cells were growing at a density of 10^5^∼10^6^ cells per well in a six-well plate. The next day, cells were transfected with CCN1 overexpression plasmid or CCN1 interference RNA. At 48 h post-transfection, cells were infected and incubated with PEDV for 24 h. After 24 h, cells were harvested and stained using annexin V/fluorescein isothiocyanate (FITC) apoptosis detection kit (BioVision, Inc., Milpitas, CA, United States) and analyzed by flow cytometry (Becton Dickinson, New York, NY, United States).

### Cell Viability Assay

Marc-145 cells were seeded into 96-well plate and added different concentration of signal pathway inhibitor when density was about 90%. Cells were incubated at 37°C for 48 h, and then, 20 μl of 3-(4,5-Dimethylthiazol-2-yl)-2,5-diphenyltetrazolium bromide (MTT) labeling reagent was added to each well and addition incubated for 4 h. After adding 150 μl of solubilizing solution, the absorbance at 550 nm using an enzyme-linked immunosorbent assay reader was measured.

### Statistical Analysis

All experiments were performed with at least three independent experiments, group data were presented as means ± SD and were assessed using one-way ANOVA. For each assay, Student’s *t*-tests were used to assess differences in GraphPad Prism software. Significance difference was denoted in the figures as follows: *p ≤ 0.05, ^**^P ≤ 0.01, and ^***^p ≤ 0.001.

## Results

### The Replication of Porcine Epidemic Diarrhea Virus in Marc-145 Cells

To determine the propagation kinetics of PEDV in Marc-145 cells, the mRNA levels and TCID_50_ of PEDV infection at different times were investigated. As shown in Supplementary Figure 1A, 24 h after PEDV infection, the N gene increased 300 times compared with the control group. Moreover, the multiplication peak of PEDV appeared at 48 h after infection, and then gradually decreased at 72 h after infection. In addition, the growth-kinetics curve showed that with the increase of infection time, the replication of PEDV was gradually enhanced (Supplementary Figure 1B), which was consistent with the results of mRNA. Together, these results indicate that PEDV can infect Marc-145 cells with high efficiency and multiply rapidly.

### Porcine Epidemic Diarrhea Virus Infection Promotes Cell Communication Network Factor 1 Expression in Marc-145

**FIGURE 1:**
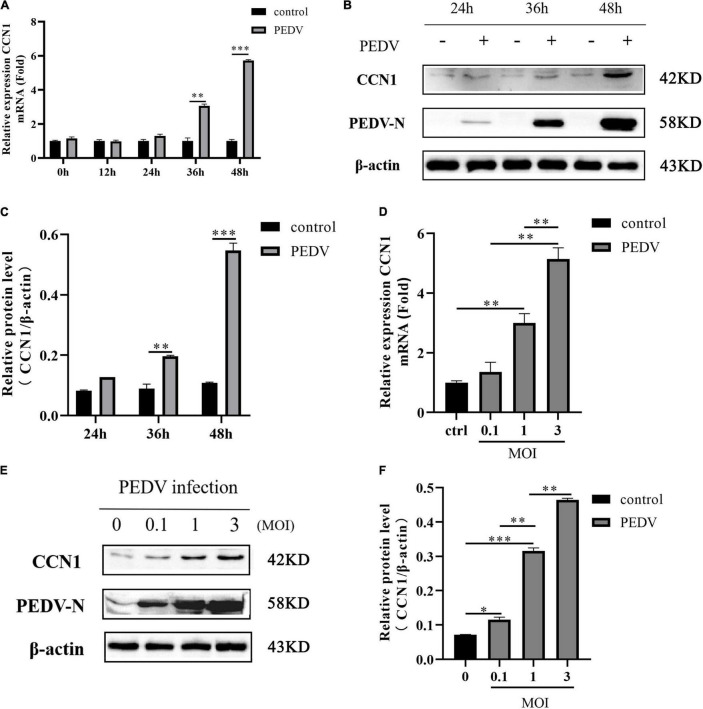
Porcine epidemic diarrhea virus infection induces CCN1 expression in Marc-145 cells. **(A,B)** Marc-145 cells were incubated with PEDV at MOI of 1, and then, the cell was harvested at indicated time point post-infection. Quantitative real-time PCR was used to detect CCN1 expression **(A)** and Western blotting was used to analyze CCN1 protein levels **(B)**. **(C)** Densitometric analysis of CCN1 relative to β-actin using ImageJ. **(D,E)** Marc-145 was infected with PEDV at MOIs of 0.1, 1, and 3 for 48 h, and the cells were collected to analyze CCN1 mRNA levels using real-time PCR **(D)** and CCN1 protein levels by Western blotting **(E)**. **(F)** Densitometric analysis of CCN1 relative to β-actin using ImageJ. The data were performed from three independent experiments. The differences were evaluated using Student’s *t*-test, and significance differences were denoted by *p < 0.05, ^**^p < 0.01, and ^***^p < 0.001.

Previous studies have determined that CCN1 expression was enhanced when infected with Zika and PRRSV (Park and Chun, 2020; Sun et al., 2020). However, there are few study reports concerning PEDV regulating CCN1 expression in Marc-145 cells. To determine whether the expression of CCN1 was influenced by PEDV, Marc-145 was infected with PEDV and harvested at different times after infection. Then, qRT-PCR and Western blot were used to analyze the expression levels of CCN1 mRNA and protein. The results showed that CCN1 mRNA was elevated gradually at 36 and 48 h after infection (Figure 1A); CCN1 protein expression was also increased significantly at 36 and 48 h after PEDV infection (Figures 1B,C). Then, the dose dependency of the expression of CCN1 on PEDV was verified. Marc-145 was infected with PEDV at MOIs of 0.1, 1, or 3. The data indicate that the elevation of CCN1 mRNA (Figure 1D) and protein (Figures 1E,F) by PEDV was in a dose-dependent manner. In addition, we confirm the results in other PEDV-susceptible cell lines. We performed similar experiments using Vero cells. The mRNA and protein levels were detected similar results in PEDV-infected Vero cells (Supplementary Figure 2). These results suggested that PEDV upregulated the expression of CCN1 at the transcriptional levels and protein levels.

### Exploring the Potential Transcription Factor Binding Sequence for the Activation of Cell Communication Network Factor 1 Promoter Activity

**FIGURE 2:**
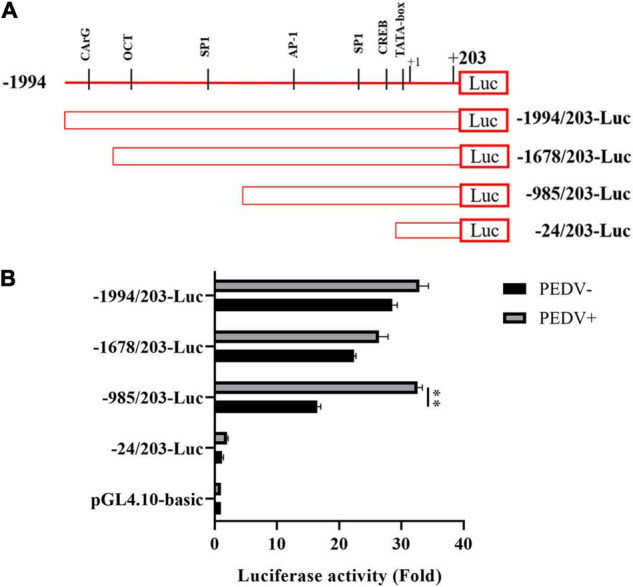
Cell communication network factor 1 promoter sequences were cloned, analyzed, and mapped latent PEDV-responsive regulatory elements. **(A)** Schematic representation of the CCN1 promoter and CCN1 promoter truncated mutants were inserted into pGL4.10-basic vectors, and the constructed vectors were denoted as -1994/203-Luc, -1678/203-Luc, -985/203-Luc, and -24/203-Luc. **(B)** Marc-145 cells were respectively transfected with a variety of constructed plasmid for 24 h and incubated with PEDV. At 48 h post-infection, cells were collected and detected the luciferase activity. The data were performed from three independent experiments. (PEDV−, mock infection group; PEDV+, PEDV infection group). The differences were evaluated using Student’s *t*-test, and significance differences were denoted by ^**^p < 0.01.

To further decipher the transcriptional regulation mechanism of PEDV-induced CCN1 production, the 5′ flanking 2,197-bp regions of the CCN1 gene were cloned. Using bioinformatics approach (Promoter 2.0 and ALGGEN prediction programs) analysis shows that several underlying transcriptional regulatory elements, including CArG (−1,937∼−1,946), OCT (−1,634∼−1,644), and SP1 (−1,211∼−1,221; −273∼−283), AP-1 (−811∼−823), and CREB (−40∼−51), were identified in the CCN1 promoter. To assess the CCN1 promoter activity and find the regions of the CCN1 promoter that respond to PEDV stimulation, a series of CCN1 promoter 5′ flanking region truncation sequences were constructed, cloned, and inserted into a pGL4.10-basic reporter vector (Figure 2A). Marc-145 cells were transfected with 1 μg of a variety of constructed plasmid and 0.2 μg of pRL-TK plasmid for 24 h, and then, the cell was infected with or without the PEDV. Luciferase assay showed that all truncated sequences had higher luciferase activities with or without PEDV stimulation compared to the pGL-4.10 basic, except the construct −24/203-luc (Figure 2B). This result indicated that all the constructs have promoter activity, among the –985/203-luc mutants exhibited about 1.5-fold upregulation after PEDV infection. However, the –24/203-luc mutants had no significant difference after PEDV stimulation. Therefore, the –985/203 region of the CCN1 promoter may contain transcription factor binding sites in response to PEDV infection.

### AP-1 and CREB Is Essential for Porcine Epidemic Diarrhea Virus to Activate the Cell Communication Network Factor 1 Promoter

**FIGURE 3:**
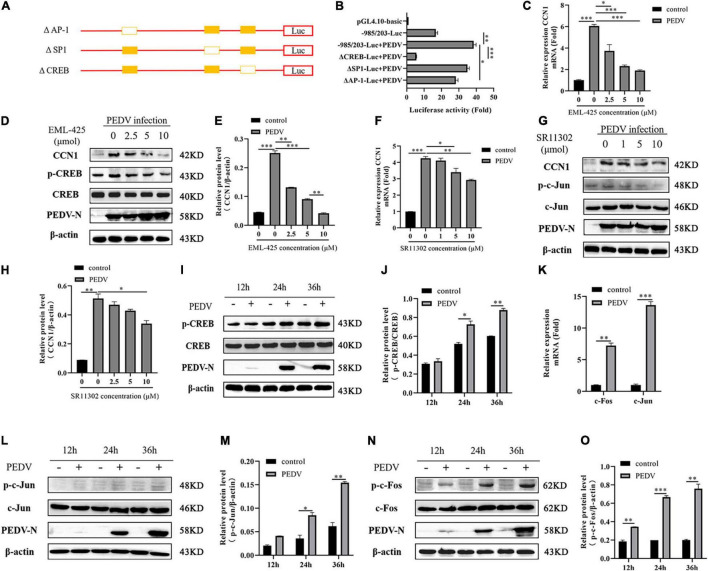
The CREB binding site and AP-1 binding site are indispensable for CCN1 production. **(A)** Schematic diagram represents the –985/203-luc CCN1 promoter deletion mutant vectors. **(B)** The –985/203-luc CCN1 promoter and variety of deletion mutant were transfected into Marc-145 cells for 24 h. The cells were infected with PEDV for 48 h determining the luciferase activity. **(C,D)** Marc-145 cells were treatment using EML-425 (CREB inhibitor) with different concentrations at 1 h and then using PEDV incubated for 48 h. Real-time PCR was used to analyze CCN1 levels **(C)**, and Western blotting was used to analyze the protein expression of CCN1 **(D)**. **(E)** Densitometric analysis of CCN1 relative to β-actin using ImageJ. **(F,G)** Marc-145 cells were treatment using SR11302 (AP-1 inhibitor) at 1 h and then using PEDV incubated for 48 h. Real-time PCR was used to analyze CCN1 levels **(F)**, and Western blotting was used to analyze the protein expression of CCN1 **(G)**. **(H)** Densitometric analysis of CCN1 relative to β-actin using ImageJ. **(I)** Marc-145 cells were incubated with PEDV, and cells were collected at 12, 24, and 36 h. Western blotting was used to examine the level of CREB and phospho-CREB. **(J)** Densitometric analysis of phospho-CREB relative to CREB using ImageJ. **(K)** Marc-145 cells were infected with PEDV, cells were harvested at 48 h, and the mRNA level of c-Jun and c-Fos were analyzed using real-time PCR. **(L)** Marc-145 cells were infected with PEDV, and cells were harvested at 12, 24, and 36 h. Western blotting was used to examine the level of c-Jun and phospho-c-Jun. **(M)** Densitometric analysis of phospho-c-Jun relative to β-actin using ImageJ. **(N)** Marc-145 cells were infected with PEDV, and cells were harvested at 12, 24, and 36 h. Western blotting was used to examine the level of c-Fos and phospho-c-Fos. **(O)** Densitometric analysis of phospho-c-Fos relative to β-actin using ImageJ. The data were performed from three independent experiments. The differences were evaluated using Student’s *t*-test, and significance differences were denoted by *p < 0.05, ^**^p < 0.01, ^***^p < 0.001.

Three transcription factor binding sites were found in the −985 to 203 regions of the CCN1 promoter: AP-1 binding site (−811∼−823), Sp1 binding site (−1,211∼−1221), and CREB binding site (−40∼−51). To identify specific transcription factors in the CCN1 promoter, which respond to PEDV infection, three deletion mutants of transcription factor binding sites were constructed (Figure 3A). Then, luciferase analysis was used to detect the activity of the mutated CCN1 promoter. The result showed that the deletion of AP-1 and CREB binding sites dramatically impaired the activity of the CCN1 promoter (Figure 3B). Moreover, the effect of the CREB binding site is relatively more significant. To further prove that AP-1 and CREB binding sites mediate transcription induction of the CCN1 promoter, SR11302 (AP-1 inhibitor) or EML-425 (CREB inhibitor) was used to block the activity of AP-1 or CREB. The cytotoxicity of different concentration inhibitors was detected by a cell viability assay (Supplementary Figures 3A,B). The obtained results are consistent with the analysis of Luciferase report. EML-425 notably inhibited PEDV-induced CCN1 mRNA (Figure 3C) and protein (Figures 3D,E) expression in a dose-dependent manner. However, the difference of CCN1 mRNA expression was exhibited when the concentration of SR11032 was 5 and 10 μM (Figure 3F), and CCN1 protein levels were suppressed significantly with 10 μM SR11302 treatment (Figures 3G,H).

To determine whether AP-1 and CREB pathways were activated by PEDV, the phosphorylation levels of AP-1 and CREB were detected. Western blot analysis suggested that the level of CREB phosphorylation was elevated with the increase of infection time (Figures 3I,J). AP-1 is a dimeric complex, which consists of c-Jun and c-Fos. By detecting the transcription expression of c-Jun and c-Fos, it was determined that the mRNA levels of c-Jun and c-Fos increased significantly after PEDV infection (Figure 3K), and then, we detect the level of c-Jun and c-Fos protein. As shown in Figures 3L,M, PEDV infection promoted the expression of c-Jun phosphorylation, and there was a slight increase compared with a mock group at 24 and 36 h post-infection. The level of c-Fos phosphorylation is elevated significantly after PEDV infection (Figures 3N,O). Together, all data demonstrated that PEDV infection promotes the expression of CCN1 through AP-1 and CREB binding elements.

### PKA and p38 Signaling Pathways Are Involved in Porcine Epidemic Diarrhea Virus-Induced Cell Communication Network Factor 1 Expression

**FIGURE 4:**
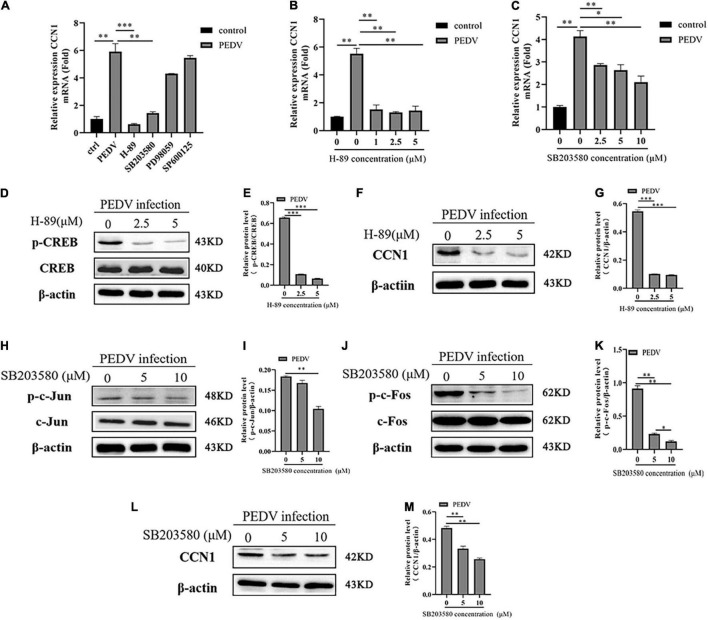
The PKA and P38 pathways are involved in PEDV-mediated the expression of CCN1. **(A)** Marc-145 cells were pretreated using H-89, SB203580, PD98059, and SP600125 for 1 h then infected with PEDV for 48 h, real-time PCR analyzed the mRNA expression of CCN1. **(B)** Marc-145 cells were pretreated using H-89 at doses of 1, 2.5, and 5 for 1 h and then infected with PEDV 48 h; real-time PCR analyzed the mRNA expression of CCN1. **(C)** Marc-145 cells were pretreated using SB203580 at doses of 2.5, 5, and 10 for 1 h and then infected with PEDV for 48 h; real-time PCR analyzed the mRNA expression of CCN1. **(D)** Marc-145 cells were pretreated using H-89 at doses of 0, 2.5, and 5 for 1 h and then infected with PEDV for 48 h; Western blotting assessed the expression level of CREB and phospho-CREB. **(E)** Densitometric analysis of phospho-CREB relative to CREB using ImageJ. **(F)** Marc-145 cells were pretreated using H-89 at doses of 0, 2.5, and 5 μM for 1 h and then infected with PEDV for 48 h; Western blotting assessed the expression level of CCN1. **(G)** Densitometric analysis of CCN1 relative to β-actin using ImageJ. **(H)** Marc-145 cells were pretreated using SB203580 at doses of 0, 5, and 10 μM for 1 h and then infected with PEDV for 48 h; Western blotting assessed the level of c-Jun and phospho-c-Jun. **(I)** Densitometric analysis of phospho-c-Jun relative to β-actin using ImageJ. **(J)** Marc-145 cells were pretreated using SB203580 at doses of 0, 5, and 10 μM for 1 h and then infected with PEDV for 48 h; Western blotting assessed the level of c-Fos and phospho-c-Fos. **(K)** Densitometric analysis of phospho-c-Fos relative to β-actin using ImageJ. **(L)** Marc-145 cells were pretreated using SB203580 at doses of 0, 5, and 10 μM for 1 h and then infected with PEDV for 48 h; Western blotting assessed the level of CCN1. **(M)** Densitometric analysis of CCN1 relative to β-actin using ImageJ. The data were performed from three independent experiments. The differences were evaluated using Student’s *t*-test, and significance differences were denoted by *p < 0.05, ^**^p < 0.01, and ^***^p < 0.001.

To dissert the upstream signal molecules that PEDV regulates the expression of CCN1, Marc-145 using inhibitors of the signaling pathways, including PKA, p38, JNK, ERK1/2, were incubated for 1 h and then infected with PEDV. At 48 h post-infection, CCN1 expression was analyzed by RT-PCR. As shown in Figure 4A, PEDV-induced CCN1 expression was decreased significantly by the addition of SB203580 (p38 inhibitor) and H-89 (PKA inhibitor), whereas the SP600125 (JNK inhibitor) and PD98059 (ERK1/2 inhibitor) did not affect PEDV-induced CCN1 expression. To further confirm the above results, different concentrations of inhibitors were added to Marc-145 before infecting with PEDV for 48 h. The H-89 (PKA inhibitor) and SB203580 (p38 inhibitor) significantly inhibited the production of CCN1 mRNA induced by PEDV (Figures 4B,C). These data suggest that the activation of CCN1 is mediated by PKA and p38 pathways. The concentration of different pathways inhibitors did not affect cell viability (Supplementary Figure 3C).

Next, the correlation between PKA, p38, and CREB, AP-1 was investigated during PEDV infection. Marc-145 cells were treated with H-89 (PKA inhibitor) and SB203580 (p38 inhibitor) at different concentrations for 1 h before PEDV infection. At 48 h post-infection, cells were harvested to assess the level of CCN1, phospho-CREB, phospho-c-Jun, and phospho-c-Fos using Western blotting. The results showed that H-89 (PKA inhibitor) dramatically impaired PEDV-induced CREB phosphorylation levels in a dose-dependent manner (Figures 4D,E), and the expression of CCN1 protein was also suppressed with the increase of the concentration of H-89 (Figures 4F,G). The addition of SB203580 (p38 inhibitor) significantly inhibited the c-Jun and c-Fos phosphorylation levels (Figures 4H–K). Moreover, CCN1 protein production is also decreased significantly when using the treatment of SB203580 (Figures 4L,M). Together, these observations indicate that p38 and PKA signaling pathways are involved in PEDV-mediated induction of CCN1.

### Effect of Cell Communication Network Factor 1 on Porcine Epidemic Diarrhea Virus Replication

**FIGURE 5:**
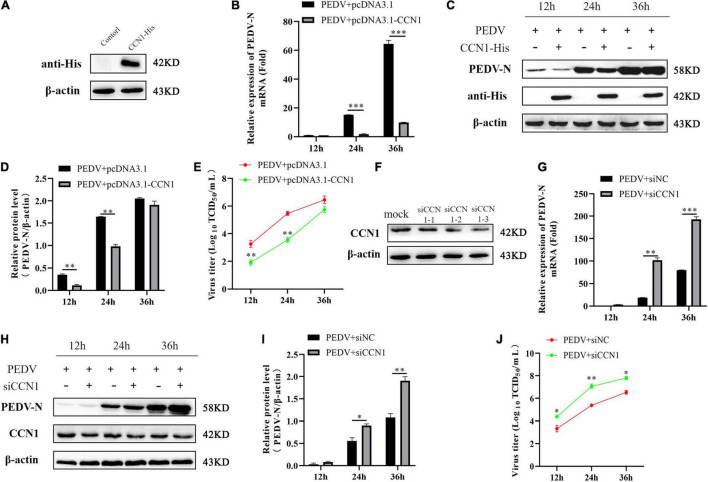
The CCN1 protein to the effect of PEDV replication. **(A)** Marc-145 cells were transfected with pcDNA3.1-CCN1-his or pcDNA3.1-his vector for 36 h. Western blot detected CCN1 expression efficiency. **(B–D)** Marc-145 cells were transfected with pcDNA3.1-CCN1-his or pcDNA3.1-his vector for 36 h. PEDV at MOI 1 was used to infect cell for 1 h. Then, cells and supernatant were harvested to evaluate the mRNA and protein expression level of PEDV-N by real-time PCR **(B)**, Western blot **(C)**, and densitometric analysis **(D)**. **(E)** The supernatant was harvested to assess viral title by TCID_50_. **(F)** Marc-145 cells were transfected with three siRNA for 48 h, and CCN1 knockdown efficiency was assessed. **(G–J)** Marc-145 cells were transfected with siNC or siCCN1 for 48 h, and cells were incubated with PEDV at MOI 1 for 1 h. Then, using real-time PCR and Western blot, respectively, detected PEDV-N mRNA **(G)**, protein expression **(H)**, and densitometric analysis **(I)**. TCID_50_ was used to examine viral title **(J)**. The data were performed from three independent experiments. The data were performed from three independent experiments. The differences were evaluated using Student’s *t*-test, and significance differences were denoted by *p < 0.05, ^**^p < 0.01, and ^***^p < 0.001.

The effect of CCN1 on the virus has rarely been reported. Because PEDV induces significant expression of CCN1 mRNA and protein in Marc-145, we hypothesized that CCN1 might play a crucial role in PEDV replication. To verify the above hypothesis, the pcDNA3.1-his and pcDNA3.1-CCN1-his were transfected, respectively, into Marc-145 and infected with PEDV. As presented in Figure 5A, CCN1 can be expressed effectively in Marc-145 cells. As expected, the results showed that compared with the empty vector group, the overexpression of CCN1 significantly downregulated the level of PEDV-N mRNA (Figure 5B). In particular, the expression of PEDV-N protein was significantly reduced at 24 h (Figures 5C,D). The virus titers were measured by collecting cell supernatants. The data show that it is consistent with the results of the mRNA and protein (Figure 5E). To further verify the relevance of CCN1 in PEDV proliferation, we generated three special siRNAs targeting CCN1. Western blot was performed to assess the interfere efficiency of CCN1 in Marc-145. As presented in Figure 5F, siCCN1-3 exhibited a more obvious knockdown efficiency than siCCN1-1 and siCCN1-2. The following experiments were carried out with siCCN1-3. The results showed that CCN1 interference facilitates the expression of PEDV-N mRNA and protein (Figures 5G–I) and increased virus titer (Figure 5J). Together, the overexpression of CCN1 inhibited the replication of PEDV, whereas interference of CCN1 facilitated the replication of PEDV.

### The Effect of Cell Communication Network Factor 1 in Suppressing Porcine Epidemic Diarrhea Virus Does Not Depend on the Expression of Interferon

**FIGURE 6:**
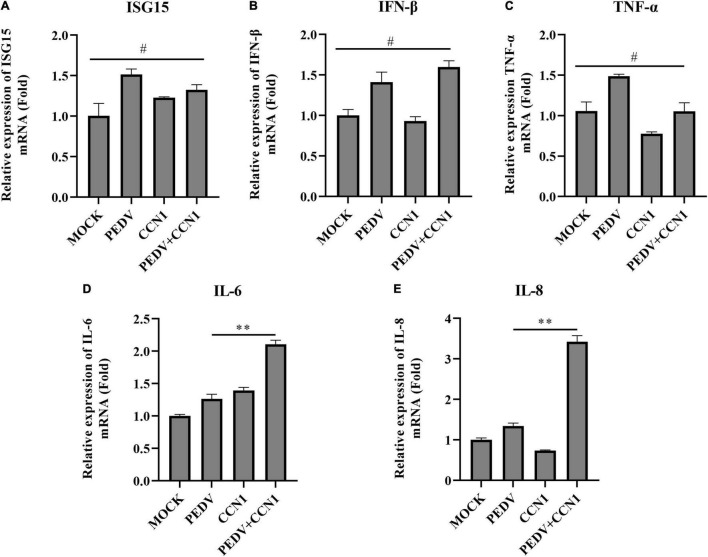
Cell communication network factor 1 has no effect on the expression of interferon. **(A–E)** Marc-145 cells were transfected with pcDNA3.1-CCN1-his or pcDNA3.1-his vector for 36 h and were infected with or without the PEDV strain. At 24 h, cells were collected to analyze the mRNA level of ISG15 **(A)**, IFN-β **(B)**, TNF-α **(C)**, IL-6 **(D)**, and IL-8 **(E)** by real-time PCR assay. The data were performed from three independent experiments. The differences were evaluated using Student’s *t*-test, and significance differences were denoted by ^**^p < 0.01.

The above study found that CCN1 overexpression decreases PEDV replication. To study whether CCN1 exerts antiviral function on Marc-145 cells by producing inflammatory cytokines and type I interferon. The CCN1 overexpression plasmids were transfected into Marc-145 cells. The data showed that overexpression of CCN1 promotes PEDV-induced IL-6 (Figure 6D) and IL-8 (Figure 6E) mRNA levels, whereas did not affect the mRNA expression of ISG15 ubiquitin-like modifier (ISG15) (Figure 6A), interferon β (IFN-β) (Figure 6B), and tumor necrosis factor α (TNF-α) (Figure 6C). It is widely known that IFN-β has a direct antiviral function, but CCN1 has not changed the expression of IFN-β, suggesting that CCN1 suppresses the replication of PEDV maybe through other pathways. Together, the results indicated that the effect of CCN1 in suppressing PEDV does not depend on the expression of interferon.

### Cell Communication Network Factor 1 Regulates Porcine Epidemic Diarrhea Virus-Induced Apoptosis

**FIGURE 7:**
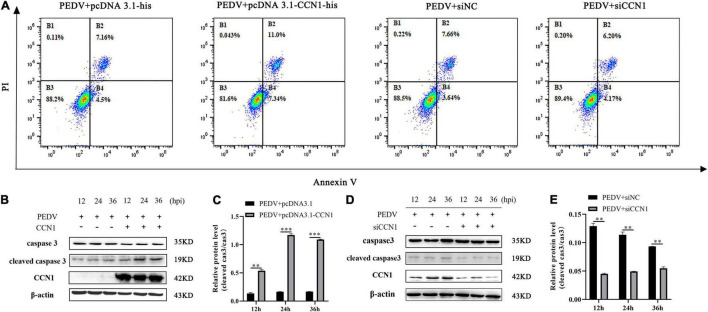
Cell communication network factor 1 regulates PEDV-induced apoptosis in Marc-145 cells. **(A)** Marc-145 cells were transfected with pcDNA3.1-his, pcDNA3.1-CCN1-his, siNC, and siCCN1, stained with annexin V and PI, and apoptosis was detected by flow cytometry. **(B)** Marc-145 cells were transfected with pcDNA3.1-CCN1-his or pcDNA3.1-his vector for 36 h and infected with the PEDV. Cells were harvested at the indicated time, and Western blot was used to assess the expression of the caspase 3 and cleaved caspase 3. **(C)** Densitometric analysis of cleaved caspase 3 relative to caspase 3 using ImageJ. **(D)** Marc-145 cells were transfected with siNC or siCCN1 for 48 h and infected with the PEDV. Cells were harvested at the indicated time, and Western blot was used to assess the expression of the caspase 3 and cleaved caspase 3. **(E)** Densitometric analysis of cleaved caspase 3 relative to caspase 3 using ImageJ. The data were performed from three independent experiments. The differences were evaluated using Student’s *t*-test, and significance differences were denoted by ^**^p < 0.01, ^***^p < 0.001.

It has previously been reported that CCN1 through (TNF)-related apoptosis-inducing ligand (TRAIL) induces EAC cell apoptosis (Dang and Chai, 2020). To study whether CCN1 is involved in PEDV-induced apoptosis, CCN1 overexpression plasmids and siCCN1 were transfected in Marc-145. At 24 h post-transfection, cells were infected with PEDV and the apoptosis of Marc-145 cells was evaluated by flow cytometry. Compared with the empty vector, the apoptotic rate in Marc-145 cells with CCN1 overexpression was notably increased. Moreover, the apoptotic proportion in Marc-145 cells with the CCN1 knockdown vector decreased compared with the negative control cells (Figure 7A). To confirm the above results, using Western blot analysis, the cleaved caspase 3 protein levels revealed that, compared with the empty vector group, the cleaved caspase 3 increased in the CCN1 overexpression group (Figures 7B,C). Instead, the knockdown of CCN1 attenuated caspase 3 cleavage (Figures 7D,E). The results indicate that CCN1 can regulate PEDV-induced apoptosis in Marc-145 cells.

### Cell Communication Network Factor 1 Regulated Porcine Epidemic Diarrhea Virus-Induced Apoptosis Through the Mitochondrial Pathway

**FIGURE 8:**
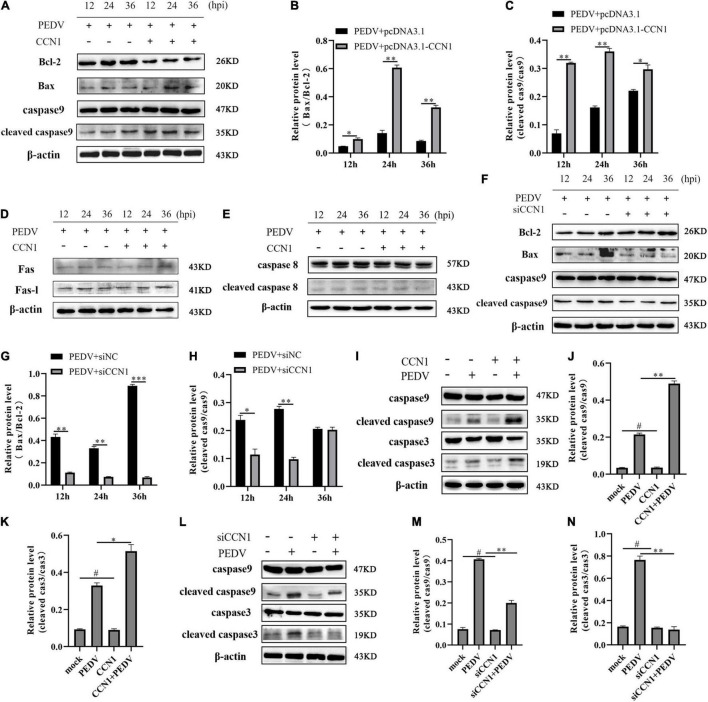
The mitochondrial pathway was involved CCN1 induced apoptosis. **(A–C)** Marc-145 cells were transfected with pcDNA3.1-CCN1-his or pcDNA3.1-his vector for 36 h and infected with the PEDV. Cells were harvested at the indicated time, Western blot was used to assess the expression of the Bcl-2, Bax, caspase 9, and cleaved caspase 9 **(A)**, densitometric analysis of Bax relative to Bcl-2, and cleaved caspase 9 relative to caspase 9 using ImageJ **(B,C)**. Western blot was used to elevate the level of the Fas-L, Fas, caspase 8, and cleaved caspase 8 **(D,E)**. **(F–H)** Marc-145 cells were transfected with siNC or siCCN1 for 48 h and infected with the PEDV. Cells were harvested at the indicated time, Western blot was used to assess the expression of the Bcl-2, Bax, caspase 9, and cleaved caspase 9 **(F)**, densitometric analysis of Bax relative to Bcl-2, and cleaved caspase 9 relative to caspase 9 using ImageJ **(G,H)**. **(I–K)** Marc-145 cells were transfected with pcDNA3.1-CCN1-his or pcDNA3.1-his vector for 36 h and infected with the PEDV. Cells were harvested at 24 h after PEDV infection, Western blot was used to assess the expression of the caspase 9, cleaved caspase 9, caspase 3, and cleaved caspase 3 **(I)**, densitometric analysis of cleaved caspase 9 relative to caspase 9, and cleaved caspase 3 relative to caspase 3 using ImageJ **(J,K)**. **(L–N)** Marc-145 cells were transfected with siNC or siCCN1 for 48 h and infected with the PEDV. Cells were harvested at 24 h after PEDV infection, and Western blot was used to assess the expression of the caspase 9, cleaved caspase 9, caspase 3, and cleaved caspase 3 **(L)**, densitometric analysis of cleaved caspase 9 relative to caspase 9, and cleaved caspase 3 relative to caspase 3 using ImageJ. **(M,N)** The data were performed from three independent experiments. The differences were evaluated using Student’s *t*-test, and significance differences were denoted by *p < 0.05, ^**^p < 0.01, and ^***^p < 0.001.

To gain the specific mechanism of CCN1 involved in the apoptosis induced by PEDV. We measured the protein-related death receptor pathway (Fas/FasL/caspase 8) and the protein-related of the mitochondrial pathway (Bcl-2/Bax/caspase 9). The result showed that overexpression of CCN1 resulted in anti-apoptotic protein Bcl-2 decreased compared with the empty vector group, and pro-apoptotic protein Bax expresses levels increased (Figures 8A,B). Moreover, cleaved caspase 9 increased in the CCN1 overexpression groups (Figures 8A,C). However, the death receptor-related proteins Fas, FasL, and caspase 8 have no significant difference in pcDNA3.1-CCN1-his–transfected cells (Figures 8D,E). Then, CCN1 knockdown was used to investigate Bax, Bcl-2, and caspase 9. The data showed that knockdown of CCN1 decreased the activation of Bax and caspase 9 and increased the expression of Bcl-2 (Figures 8F–H). Further studying whether the CCN1 protein itself can induce cell apoptosis, the results show that, when CCN1 is overexpressed or knocked down, it has no effect on the lysis of caspase 3 and caspase 9, and only under the stimulation of the virus, CCN1 can play a role (Figures 8I–N). Together, these results showed that CCN1 regulates PEDV-induced apoptosis by regulating the level of Bcl-2 and Bax to activate caspase 9.

### Cell Communication Network Factor 1 Regulated Mitochondrial-Mediated Apoptosis Through p53-Dependent Pathway to Inhibit Porcine Epidemic Diarrhea Virus Replication

**FIGURE 9:**
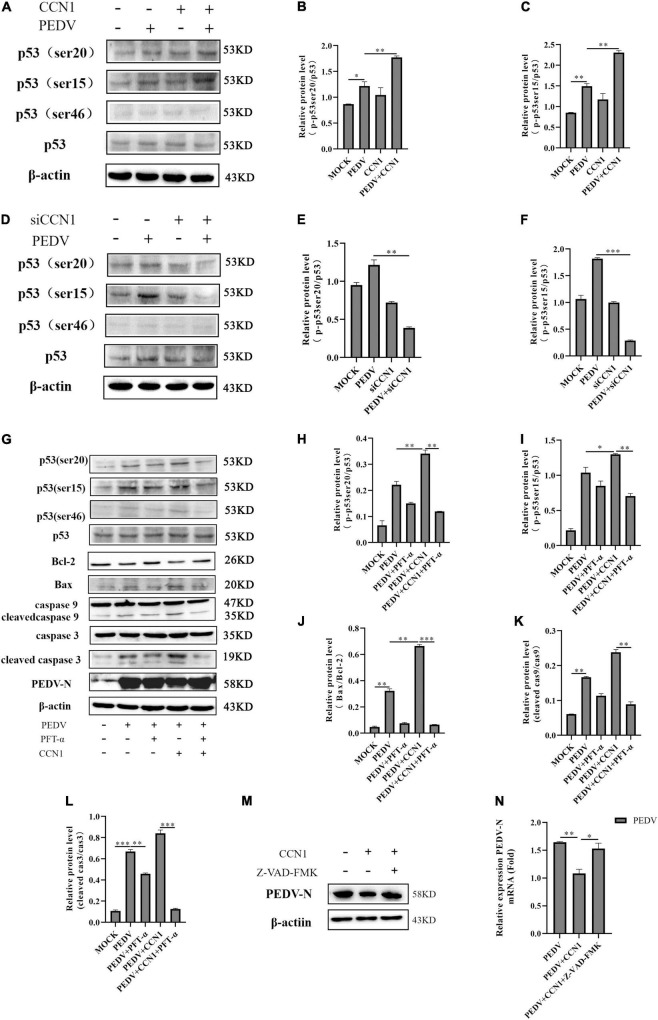
The role of p53 in CCN1-mediated apoptosis. **(A–C)** Marc-145 cells were transfected with pcDNA3.1-CCN1-his or pcDNA3.1-his vector for 36 h and then with or without the PEDV strain to infect cells for 24 h; Western blot was used to detect the abundance of the p53, phospho-p53(ser15), phospho-p53(ser20), and phospho-p53(ser46). **(D–F)** Marc-145 cells were transfected with siNC or siCCN1 for 48 h and then with or without the PEDV strain to infect cells for 24 h; Western blot was used to detect the expression of the p53, phospho-p53(ser15), phospho-p53(ser20), and phospho-p53(ser46). **(G–L)** Marc-145 cells were transfected with pcDNA3.1-CCN1-his or pcDNA3.1-his vector for 36 h, using pretreated PFT-α 1 h before infecting with PEDV; Western blot analyzes caspase 9, cleaved caspase 9, caspase 3, cleaved caspase 3, Bax, Bcl-2, PEDV-N, p53, and phosphorylated p53 expression levels. **(M,N)** Marc-145 cells were transfected with pcDNA3.1-CCN1-his or pcDNA3.1-his vector for 36 h, using pretreated Z-VAD-FMK 1 h before infecting with PEDV; Western blot analyzes PEDV expression levels. The data were performed from three independent experiments. The differences were evaluated using Student’s *t*-test, and significance differences were denoted by *p < 0.05, ^**^p < 0.01, and ^***^p < 0.001.

Tumor suppressor p53 is known to be involved in the regulation of cell senescence and apoptosis (Wawryk-Gawda et al., 2014). Our previous study found that PEDV infection could induce cell apoptosis by activating the p53 signal pathway (Xu et al., 2019). To further determine whether CCN1 has an influence on the p53 pathway, Western blot was used to detect the p53 total protein levels and phosphorylated p53 protein levels. As shown in Figures 9A,D, there is no effect on the p53 total protein level of both CCN1 overexpression and CCN1 knockdown. However, the phosphorylated p53 levels had changed significantly. CCN1 overexpression catalyzed phosphorylation of p53 at serine 20 (phospho-p53 ser20) and serine 15 (phospho-p53 ser15) compared to the empty vector group (Figures 9A–C). Knockdown of CCN1 suppress phospho-p53(ser15) and phospho-p53(ser20) level (Figures 9D,E). These results suggested that there was a close relationship between CCN1 protein and p53 protein phosphorylation.

Next, we used the PFT-α (p53 inhibitor) to further study whether CCN1-mediated apoptosis is through the p53 pathway. The results showed that phosphorylate levels of p53 at ser15, ser20, and ser46 were significantly suppressed by PFT-α (Figures 9G–I). Compared with the cell without pretreatment using PFT-α inhibitors, pretreated PFT-α decreases activation of caspase 3 and caspase 9 (Figures 9K,L). Besides, the expression of Bax protein was notably inhibited, whereas the expression of Bcl-2 protein was restored (Figure 9J). Together, these data indicated that the CCN1-regulated apoptosis mainly depends on the p53 pathway. Moreover, we found that the inhibitory effect of CCN1 on PEDV-N protein expression was weakened by PFT-α. CCN1 can inhibit PEDV replication and can regulate apoptosis through p53 pathway, we hypothesis that the CCN1 suppresses PEDV replication through apoptosis. To prove the relationship, using caspase inhibitor Z-VAD-FMD to inhibit apoptosis, it was found that the inhibitory effect of CCN1 on the PEDV was weakened (Figures 9M,N). Therefore, the results showed that CCN1 inhibits PEDV through apoptosis.

## Discussion

Porcine epidemic diarrhea virus evolved a variety of strategies to promote its persistent infection in the host cell (Li et al., 2021; Su et al., 2021; Xu et al., 2021). To escape the invasion of the virus, the secretion of antiviral proteins in the host cell was enhanced (Li et al., 2019; Pandey et al., 2019; Wang et al., 2021). CCN1, as a pro-inflammation factor, is regulated by many factors, including cytokines, viruses, and bacteria (Todorovic et al., 2005; Chen and Du, 2007; Kurozumi et al., 2008). It is reported that many viral infections can enhance the expression of CCN1. For example, Zika virus promotes the expression of CCN1 by the CaMKIIα-CREB pathway (Sun et al., 2020). Coxsackieviruses (CVB) and herpes simplex virus-1 (HSV-1) infection increase CCN1 expression in HeLa cell and rat glioma cells (Kim et al., 2004; Kurozumi et al., 2008). In this study, we demonstrated PEDV infection can promote the secretion of CCN1. We speculate that the expression of CCN1 may be related to the proliferation stage of the virus. The virus proliferation curve showed that the logarithmic growth phase of the virus was 36–48 h after infection. The host cell initiates defense by perceiving a large number of replicated viruses and promotes the induction of protein CCN1.

Cell communication network factor 1 promoter sequences contain a variety of regulatory elements, including CArG, OCT, AP-1, CREB, and SP1 (Kim et al., 2003; Qin et al., 2014; Seefried et al., 2017). Phosphorylated CREB can directly bind to the CCN1 promoters to enhance CCN1 transcriptional expression (Dou et al., 2017). EGF stimulation enhances CREB phosphorylation and activates the expression of CCN1 promoter (Chin et al., 2016). Nuclear translocation of myocardial protein-related transcription factor -A (promotes the expression of CCN 1 by binding to CArG box of CCN 1 promoter (Caires et al., 2007). In addition, the deletion of the AP-1 binding site reduces the activity of the CCN1 promoter by 50% (You et al., 2010). In the study, we confirmed that AP-1 and CREB were essential for the induction of CCN1, and the deletion of CREB and AP-1 binding elements decreased the expression of CCN1. According to previous reports, CREB phosphorylation depends on mitogen- and stress-activated kinases1/2, Ca^2+^–calmodulin-dependent kinase II (CaMKII), and cAMP-dependent protein kinase (PKA) (De Cesare et al., 1999). CaMKII regulates the expression of CCN1 by regulating CREB phosphorylation has been reported (Sun et al., 2020). In PKA^–/–^ cells, CCN1 promoter activity was not detected (Meyuhas et al., 2008). Thus, we explored whether PKA affects CCN1 expression through regulating CREB. We found that the addition of H-89 (PKA inhibitor) can inhibit about 80% of the mRNA expression of CCN1. The mitogen-activated protein kinase (MAPK) pathway–related protein to regulate CCN1 expression has also been studied. For example, CVB3 enhances CCN1 expression through JNK activation (Kim et al., 2004), Beas2B cells were treated by cigarette smoke extract (CSE)-induced JNK and p38 MAPK phosphorylation–induced CCN1 secretion (Moon et al., 2013), and PRRSV infection suppresses CCN1 transcription by blocking the ERK-AP-1 axis (Park and Chun, 2020). Here, our data show that the addition of SB203580 (p38 inhibitor) can suppress about 50% of the mRNA expression of CCN1 when the concentration of SB203580 was 10 μM, indicating that the PKA and p38 pathways are essential for PEDV-induced CCN1 expression.

Previous research shows that CCN1 plays a role in the replication of many viruses. For example, CCN1 could activate type I IFN antiviral defense response in glioblastoma cells, which is conducive to virus clearance (Thorne et al., 2014). However, Zika-induced CCN1 expression can promote virus replication in human astrocytoma cells (CCF-STTG1) cells (Sun et al., 2020). The current study found that the overexpression of CCN1 inhibited PEDV replication and the knockdown of CCN1 enhanced PEDV replication. The previous investigation reported that CCN1 could induce IL-8 (Wu et al., 2017) and CCL20 induction in keratinocytes (Li et al., 2017), and increased IL-6 expression level in 16HBE cells (Shi et al., 2018). Our results showed that the overexpression of CCN1 upregulated the mRNA expression level of IL-6 and IL-8 in Marc-145 cells during PEDV infection, whereas the mRNA level of IFN-β, ISG15, and TNF-α were not affected. In addition, the only overexpression CCN1 protein does not change the expression of the IL-6 and IL-8, indicating that PEDV infection is essential for the production of the IL-6 and IL-8 induced by CCN1. *In vitro*, IFN-β and ISG15 can directly resist viral infections (Zhao et al., 2020; Liu et al., 2021), whereas pro-inflammation cytokines IL-6 and IL-8 may not have antiviral effects on a single cell. However, pro-inflammation cytokines can play phagocytosis by recruiting macrophages and can also promote the secretion of inflammatory factors to resist the invasion of virus *in vivo*. From the above, we know that the antiviral effect induced by CCN1 may be through other means.

Apoptosis, as a physiological defense mechanism, can regulate viral propagation. However, the specific effect of apoptosis on the virus remains controversial. Some studies have shown that virus invades cells by holding the expression of apoptosis-related proteins to inhibit or delay the occurrence of apoptosis, which is beneficial to the reproduction of virus offspring (Umeshappa et al., 2010). Some studies have shown that, in viral infection, host proteins eliminate the infected cells by enhancing apoptosis and blocking the proliferation of the virus in the body (Barber, 2001). Recently, some evidence has demonstrated that extracellular matrix (ECM) is an associated protein involved in the production of cell apoptosis, rather than cell survival (Li et al., 2016). CCN1, as an ECM protein, reduces intrinsic apoptotic pathway and inhibits the expression of survivin promoting EAV cell apoptosis (Dang et al., 2018). The synergistic treatment of CCN1 and FasL induces cardiomyoblast apoptosis by disrupting caspase inhibitor XIAP (Su and Mo, 2014). The overexpression of CCN1 results in cell apoptosis by inducing ER stress in hepatic stellate cells (Borkham-Kamphorst et al., 2016). In the present study, we found that the overexpression of CCN1 enhanced caspase 3 activation, and the knockdown of CCN1 inhibited caspase 3 activation in Marc-145. Caspase 3 is an executive protein between intrinsic and extrinsic pathways apoptotic pathway (Jorgensen et al., 2017). The combination of apoptosis-related ligand FasL, TNF-α, and corresponding receptors initiates activation of extrinsic apoptosis pathways, whereas intrinsic apoptosis is mainly regulated by Bcl-2 family protein (Pan et al., 2018). For example, the IAV NP interacts with the anti-apoptosis protein clusterin (CLU), which inhibits the intrinsic pathway of apoptosis by binding to Bax protein (Tripathi et al., 2013). Our data clearly suggest that CCN1 is involved in the initiation of the intrinsic apoptosis pathway, CCN1 expression was associated with Bcl-2, Bax, and caspase 9 activation, rather than FasL, Fas, and caspase 8.

P53 protein plays an important role in apoptosis. Under normal circumstances, the p53 protein has low secretion and a short half-life. The p53 protein through phosphorylation levels increase to inhibit the ubiquitin degradation of cellular protein and promote the stability of p53 protein, thereby promoting the induction of its downstream factor involved in apoptosis (Moll and Petrenko, 2003). Phosphorylation of p53 protein exerts cell survival and apoptosis functions by regulating Bcl-2 family proteins (Wang et al., 2021). Previous studies have shown that PEDV infection induced the accumulation of reactive oxygen species (ROS) and enhanced the p53(ser20) phosphorylation and subsequently promoted cell apoptosis (Xu et al., 2019). In this study, we found that the CCN1 regulates apoptosis by promoting the phosphorylation level of phospho-p53(ser15) and phospho-p53(ser20). Moreover, the addition of PFT-α (p53 inhibitor) suppresses the Bax, Bcl-2, and cleaved caspase 9 and caspase 3 expression, confirming that the p53 pathway is essential for CCN1-induced apoptosis. In addition, our research found that PFT-α can restore the effect of CCN1 on PEDV, so apoptosis may be the way for CCN1 to inhibit virus replication.

## Conclusion

**FIGURE 10:**
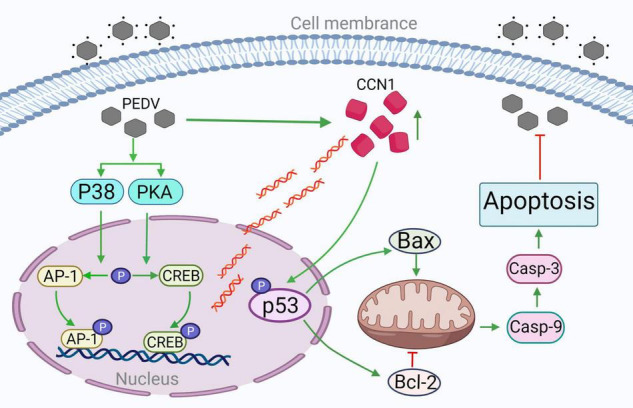
The CREB and AP-1–dependent CCN1 promote PEDV-induced apoptosis through the p53 pathway inhibiting PEDV replication. PEDV infections promote transcription factor CREB and AP-1 phosphorylation, respectively, through PKA and p38 pathways. Phosphorylated CREB and AP-1 binding to CCN1 promoter increase CCN1 expression. CCN1 overexpression increases p53 protein phosphorylation activity. The overexpression of CCN1 promotes Bax expression and inhibits Bcl-2 expression, increases the cleaved of caspase 9 and caspase 3, promotes apoptosis, and inhibits PEDV replication. The addition of p53 inhibitor decreased the occurrence of apoptosis; thus, CCN1 promotes PEDV-induced apoptosis through the p53 pathway. →, activation; ⊥, inhibition.

Our study revealed the molecular mechanism of the expression of CCN 1 in Marc-145 cells induced by PEDV and further confirmed the mechanism of CCN 1 inducing apoptosis and inhibiting the replication of PEDV. PEDV activates the transcription factors CREB and AP-1 through the PKA and p38 pathways, respectively. The activated CREB and AP-1 combine with the CCN1 promoter element to promote CCN1 expression. Then, we demonstrated that CCN1 can affect the replication of PEDV and increase pro-inflammation of the IL-6 and IL-8, rather than IFN-β, ISG15, and TNF-α. Further studies clarified that CCN1 promotes p53(ser15) and p53(ser20) phosphorylation to regulate apoptosis and inhibit PEDV replication in Marc-145 (Figure 10). Given the detriment of PEDV in the pig industry in recent years, the study possibly provides new insights into the pathogenesis mechanism of PEDV and possibly helps the development of therapeutic targets against PEDV infection.

## Data Availability Statement

The original contributions presented in the study are included in the article/Supplementary Material, further inquiries can be directed to the corresponding author/s.

## Author Contributions

HZ and YZ performed the majority of the experiments and were involved in preparation of the manuscript. JW and YY participated in the editing of the manuscript. YL and XS participated in the experimental work. QZ and XX conceived the study, participated in its design and coordination, and revised the manuscript. All authors have read and approved the final manuscript.

## Conflict of Interest

The authors declare that the research was conducted in the absence of any commercial or financial relationships that could be construed as a potential conflict of interest.

## Publisher’s Note

All claims expressed in this article are solely those of the authors and do not necessarily represent those of their affiliated organizations, or those of the publisher, the editors and the reviewers. Any product that may be evaluated in this article, or claim that may be made by its manufacturer, is not guaranteed or endorsed by the publisher.
