# Supplementary material for: The CREB and AP-1–Dependent Cell Communication Network Factor 1 Regulates Porcine Epidemic Diarrhea Virus-Induced Cell Apoptosis Inhibiting Virus Replication Through the p53 Pathway
Source: Front Microbiol. 2022 Mar 28;13:831852. doi: 10.3389/fmicb.2022.831852 (PMC8996185; doi:10.3389/fmicb.2022.831852)
Supplement: Supplementary file 1 [file Data_Sheet_1.doc]

**Supplementary Figures**


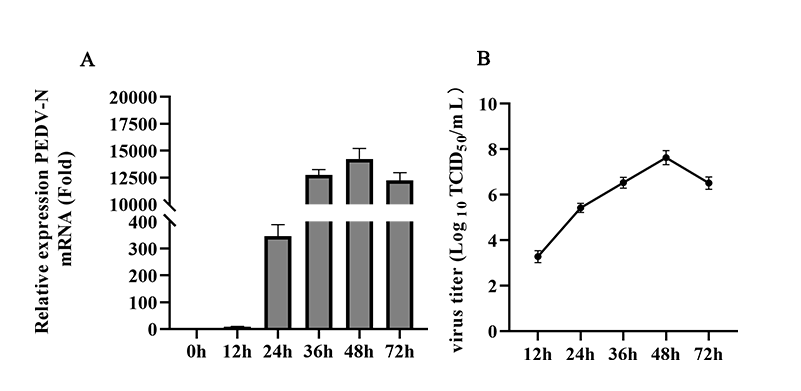
**Supplementary figure 1.** PEDV can infect the Marc-145 cells. (A) The mRNA level of PEDV-N was analyzed using Real-time PCR at indicated time point post-infection in Marc-145 cells. (B) Growth-kinetic curves were detected using TCID_50_ at indicated time point post-infection in Marc-145 cells. The data were performed from three independent experiments. The differences were evaluated using Student t-test, and significance differences was denoted by **p* < 0.05, ***p* < 0.01, ****p* < 0.001.


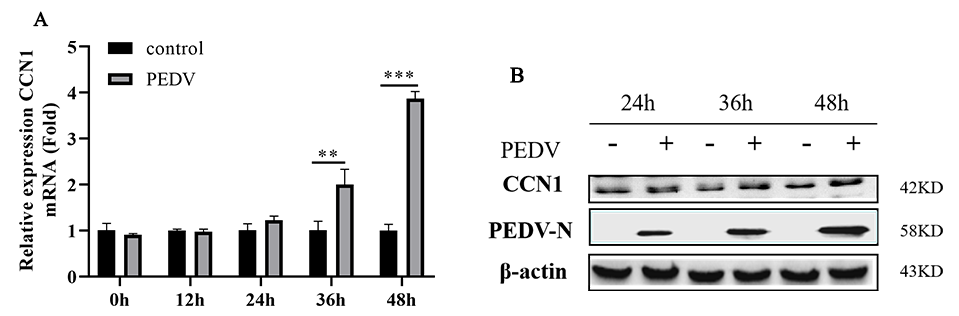


**Supplementary figure 2.** Cellular CCN1 expression level in PEDV-infected cells. Vero cells were infected with PEDV at an MOI of 1 and harvested at different times as indicated. (A) The mRNA level of PEDV-N was analyzed using real-time PCR. (B) The cell extracts were analyzed by Western blot using anti-CCN1, anti-PEDV-N, and anti-β-actin antibodies. The differences were evaluated using Student t-test, and significance differences was denoted by **p* < 0.05, ***p* < 0.01, ****p* < 0.001.


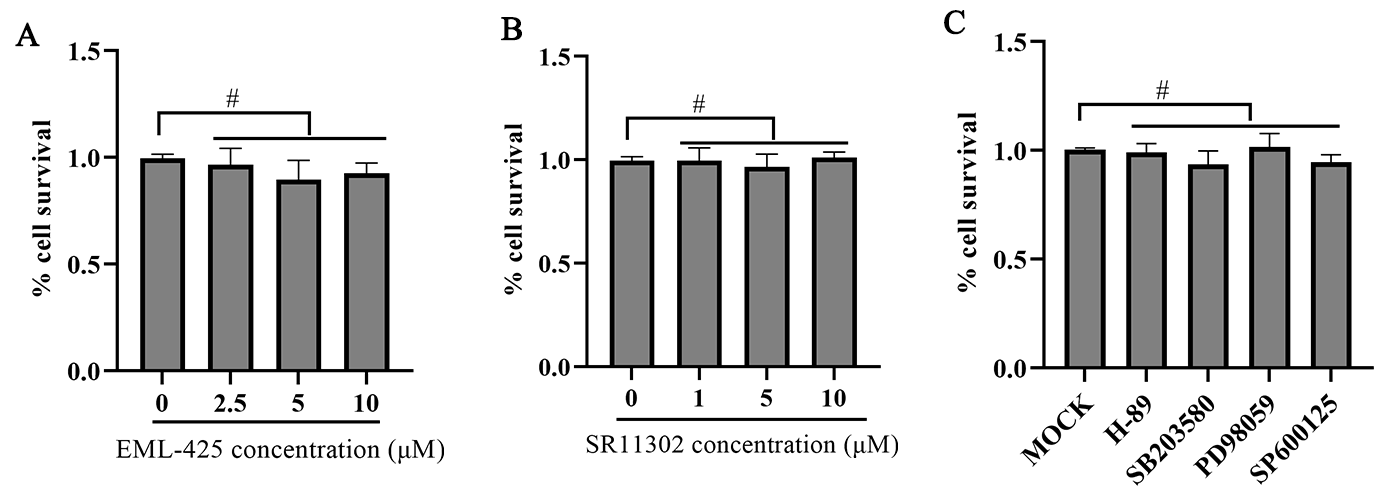


**Supplementary figure 3.** The effect of signal pathway inhibitor on cell cytotoxicity measured by MTT assay. (A) Marc-145 cells were treatment using EML-425 under 2.5, 5, and 10 μM for 48 h, cell was harvested to assess cytotoxicity. (B) Marc-145 cells were pretreated using SR11302 under 1, 5, and 10 μM for 48 h, cell was harvested to assess cytotoxicity. (C) Marc-145 cells were pretreated using pathway inhibitors (H-89, SB203580, SP600125, PD98059) for 48 h and collected cells to detect cytotoxicity. The data were performed from three independent experiments. The differences were evaluated using Student t-test, and significance differences was denoted by **p* < 0.05, ***p* < 0.01, ****p* < 0.001.
